# The Impact of Phototherapy on the Accuracy of Transcutaneous Bilirubin Measurements in Neonates: Optimal Measurement Site and Timing

**DOI:** 10.3390/diagnostics11091729

**Published:** 2021-09-20

**Authors:** Shau-Ru Ho, Yu-Chen Lin, Chi-Nien Chen

**Affiliations:** 1 Department of Pediatrics, National Taiwan University Hospital Hsin-chu Branch, Hsin-chu 30059, Taiwan; b101099017@tmu.edu.tw (S.-R.H.); yoyolin904@gmail.com (Y.-C.L.); 2 Department of Pediatrics, National Taiwan University Children’s Hospital, Taipei 10041, Taiwan; 3 Department of Pediatrics, National Taiwan University College of Medicine, Taipei 10051, Taiwan

**Keywords:** transcutaneous bilirubinometer, neonatal jaundice, phototherapy, preterm, total serum bilirubin

## Abstract

Transcutaneous bilirubinometer devices are widely applied to assess neonatal hyperbilirubinemia. However, the optimal skin site and timing of transcutaneous bilirubin (TCB) measurements for the strongest correlation with total serum bilirubin (TSB) levels after phototherapy are still unclear. We conducted a retrospective observational study evaluating the correlation of TCB and TSB levels in neonates postphototherapy. The TCB measurements on the forehead and mid-sternum at 0 and 30 min postphototherapy were assessed by using a JM-103 bilirubinometer. Paired TCB and TSB measurements were assessed by Pearson correlation and Bland–Altman plots. We analyzed 40 neonates with 96 TSB and 384 TCB measurements. The TSB level correlated moderately with the forehead TCB level at 30 min postphototherapy (*r* = 0.65) and less strongly with the midsternum TCB level at 0 min postphototherapy (*r* = 0.52). The forehead at 30 min after cessation of phototherapy was the best time point and location of TCB measurement for the assessment of neonatal jaundice status. The reliability of TCB measurements varied across skin sites and durations after phototherapy. The effectiveness of TCB measurement to assess neonatal hyperbilirubinemia is much better on covered skin areas (foreheads) 30 min postphototherapy. The appropriate application of transcutaneous bilirubinometers could aid in clinical practice and avoid unnecessary management.

## 1. Introduction

More than 50% of term and preterm neonates have neonatal hyperbilirubinemia after birth [1]. Severe hyperbilirubinemia, defined as total serum bilirubin over 20 mg/dL, affects 1.1 million infants annually [2]. Early recognition and evaluation of severe hyperbilirubinemia in these neonates are important to avoid acute bilirubin encephalopathy. Generally, the gold standard evaluation of neonatal hyperbilirubinemia and indicator of phototherapy is the measurement of serum bilirubin levels. In low-resource settings, transcutaneous bilirubin (TCB) measurement devices are an alternative option for measuring bilirubin levels [3].

TCB measurement is reliable in some studies. However, the accuracy of TCB measurement is uncertain and varies according to many factors, including preterm birth status, location of the TCB measurement, race, and concurrent phototherapy [4,5,6,7,8,9]. One meta-analysis reported a moderate correlation between TCB and total serum bilirubin TSB measurements during phototherapy [10]. The accuracy was better in the postphototherapy phase after the cessation of phototherapy.

Generally, the common locations for TCB measurement are the forehead and the sternum area. The forehead area was reported to be a better site for TCB measurement than the sternum area [11]. However, which measurement site is the most accurate is still inconclusive. TCB measurement in an area covered with skin, such as the lower abdominal area covered by a diaper or a commercialized tool, helps avoid underestimation under conditions of phototherapy [9,12,13]. For example, Rylance et al. [3] applied a bulky gauze eye mask as a covered area to detect the reliability of TCB measurements in Malawi.

Current evidence concerning the associations of the measurement locations and the time after cessation of phototherapy with the accuracy of TCB measurement is inconclusive [6,10]. Previous researchers mainly focused on the comparison of different locations or time points in separate studies, and they did not provide comprehensive results for a full data series together in one study. The intervals after stopping phototherapy were as long as 2 h [12]. However, a long waiting time may lead to insufficient phototherapy and delay the judgment of treatment. Therefore, we conducted this study to determine the optimal timing and location of TCB measurement in comparison with corresponding TSB levels in neonates after phototherapy.

## 2. Materials and Methods

### 2.1. Study Population

We conducted a retrospective observational study evaluating the correlation of TCB and TSB levels in neonates postphototherapy between December 2017 and April 2018 in the National Taiwan University Hospital Hsin-Chu branch, a regional teaching hospital with approximately 700 births annually. The decision to apply phototherapy was made based on the protocol guidelines in our institute (Table S1). During the study period, all neonates receiving phototherapy underwent TCB measurements to determine the accuracy of TSB measurements as a quality improvement project. Infants who had complete TCB and serum bilirubin measurement records were included for further analysis. Infants with congenital anomalies, previous admission for phototherapy, or exchange blood transfusions were excluded. Clinical characteristics and maternal history were also collected. The study was approved by the National Taiwan University Hospital Hsin-Chu branch Institutional Review Board and the Ethics Review Committee (109-143-E).

### 2.2. Measurement of TSB and TCB

Separate TCB measurements on the forehead (covered with a gauze eye mask; to avoid direct exposure of phototherapy to the eyes and cause injury) and mid-sternum (between nipples) at 0 and 30 min postphototherapy were recorded by using a JM-103 bilirubinometer (Draeger Medical, Telford, PA, USA) (Figure S1). In our preliminary data to examine the effect of light protection, the shading effect can reach more than 99.5% by our handmade gauze eye mask. The TCB data were recorded as the mean value of three consecutive measurements. The JM-103 transcutaneous bilirubinometer was maintained and calibrated regularly. Blood samples were acquired by the heel-stick method. Total serum bilirubin data were assessed by direct spectrophotometry (Apel BR-5200P, APEL, Kawaguchi, Japan) within 5 min after cessation of phototherapy in our central clinical laboratory.

### 2.3. Statistical Methods

The clinical characteristics of our study participants are listed. Continuous variables were summarized and are presented as the means (standard deviation, SD) or medians; categorical variables were summarized and are presented as frequencies and percentages. Pearson correlation coefficients and lines of best fit were calculated and are illustrated according to different TCB measurement locations and time points after cessation of phototherapy. We also divided differences of TCB and TSB into three groups (TCB was 2 mg/dL greater than TSB, TCB, and TSB differences were within 2 mg/dL, TCB was 2 mg/dL less than TSB) as one way for evaluating the correlation of TCB and TSB. Bland–Altman analyses were performed to evaluate the inaccuracy at each measurement location and time point after phototherapy. A p-value less than 0.05 was considered statistically significant, and these analyses were conducted using SAS 9.4 (SAS Institute, Cary, NC, USA).

## 3. Results

A total of 40 neonates with neonatal hyperbilirubinemia who met the criteria for phototherapy between December 2017 and April 2018 in the NTUH Hsin-Chu branch were included. Thirteen of them were preterm infants with a mean gestational age of 34.3 weeks. In the study participants, ninety-six measurements of TSB (total serum bilirubin) and 384 measurements of TCB (transcutaneous bilirubin) were collected. Perinatal factors, including delivery mode, maternal preeclampsia and maternal gestational diabetes, are also listed in Table 1.

The average TSB level was 12.9 mg/dL, and the correlation of the TSB levels with TCB measurements at different skin sites and time points after phototherapy are listed in Table 2. We measured TCB levels on their forehead and mid-sternum at 0 min and 30 min after cessation of phototherapy. The mean differences between TSB and TCB levels were 1.3 mg/dL, 0.9 mg/dL, 5.8 mg/dL and 5.3 mg/dL over the forehead (0 min), forehead (30 min), mid-sternum (0 min), and mid-sternum (30 min), respectively. The TSB levels correlated moderately with the forehead TCB level 30 min postphototherapy (*r* = 0.65) and were least correlated with the mid-sternum TCB level at 0 min postphototherapy cessation (*r* = 0.52). Correlations between TCB and TSB in prematurity group were higher than term neonates, especially when measurements at forehead at 30 min postphototherapy (Table 3) (*r* = 0.69).

Differences between TCB and TSB measurements are listed in Table 4. Regarding the differences in TCB and TSB, there were more measurements over the forehead (30 min) than in the other groups within ±2 mg/dL. The measurements at mid-sternum (0 and 30 min) were distributed far from TSB levels.

**Figure 1:**
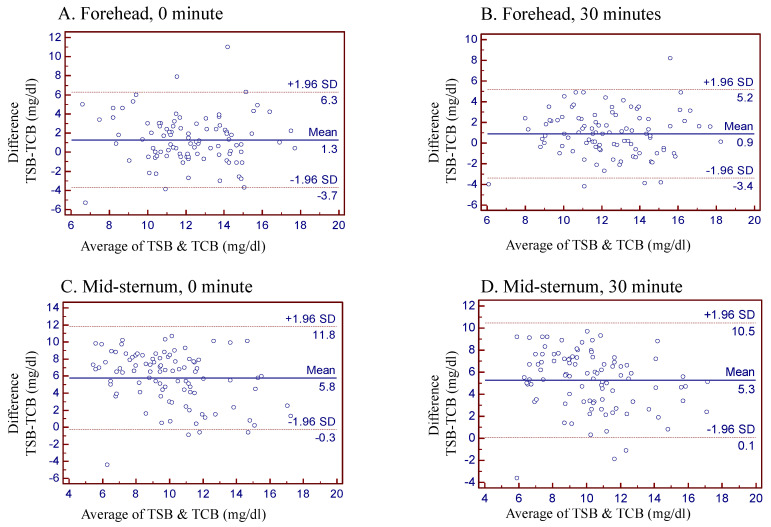
Bland–Altman plot for evaluation agreement between TCB and TSB measurements in neonates after phototherapy. (**A**) Forehead (0 min), (**B**) Forehead (30 min), (**C**) Mid-sternum (0 min) and (**D**) Mid-sternum (30 min).

Figure 1 illustrates the Bland–Altman plots describing the bias of the four different timings and areas of TCB measurements compared to the corresponding TSB data. The forehead at 30 min after cessation of phototherapy was the best time point and location of TCB measurement for the assessment of neonatal jaundice status under phototherapy treatment. The Bland–Altman plots stratified by term or preterm infants are also illustrated in the supplemental files (Figure S2: term neonates, Figure S3: preterm neonates).

## 4. Discussion

In conclusion, our study shows that TCB measurement at the forehead at 30 min after cessation of phototherapy correlated with the TSB more accurately than the other TCB measurements tested (*r* = 0.65, mean difference = 0.9 mg/dL). Our findings provide evidence demonstrating the discrepancies in TCB measurement results while assessing neonatal hyperbilirubinemia under conditions of phototherapy. Other TCB data involving the timing and location of measurements should be carefully interpreted to avoid unnecessary management and blood tests.

A transcutaneous bilirubinometer was first demonstrated in 1978 as a noninvasive tool to estimate the total serum bilirubin level via spectral reflectance of neonatal skin and subcutaneous tissue [13,14]. The common TCB measurement sites are the forehead, mid-sternum, and back. The advantages of TCB measurements are as follows: timely assessment and avoidance of the painful heel-stick method to acquire blood samples. In addition, avoidance of possible skin ecchymosis of infection. TCB measurements also reduce the frequency of serum bilirubin tests and conserve nursing manpower [15]. Previous studies have reported that transcutaneous bilirubin has a significant correlation with serum total bilirubin in term and preterm infants, even under and after phototherapy [9,15,16,17,18]. However, a discrepancy between transcutaneous and serum bilirubin measurements was still noted with extremely higher or lower bilirubin levels [19]. The factors potentially associated with inaccurate TCB results are preterm status, skin maturation condition, skin pigmentation and color, coverage of skin sites with light protection, and body sites for measurement [3,4,5,8,12,20].

With phototherapy, treatment for neonatal hyperbilirubinemia decreased bilirubin via photoisomerization, structural isomerization, and photooxidation [21]. After applying phototherapy, the correlation of TcB measurement and serum bilirubin level was decreased compared with the prior phototherapy correlation [22]. The skin bleaching effect may cause this discrepancy, and the exact timing for transcutaneous bilirubin measurement after phototherapy cessation is unknown [23]. Moreover, concurrent phototherapy may seriously impact the TCB measurement accuracy. Tan et al. [6] reported that the correlation between the TCB and TSB levels increased 18–24 h after the cessation of phototherapy. No previous study has investigated the dynamic changes in bilirubin levels during skin re-equilibration; therefore, the acceptable time interval postphototherapy for accurate assessment of TCB levels is uncertain.

Bhutani et al. [24] reported that up to 36–48 h was needed for re-equilibration of bilirubin levels postphototherapy to accurately assess TCB levels at uncovered skin sites. The long interval after discontinuing phototherapy for accurate assessment of TCB levels is inappropriate because timely bilirubin measurements are needed to initiate the appropriate therapy. Prolonged wait times and inaccuracy of the results may impede the general practice and care of vulnerable patients who need intensive phototherapy to prevent acute bilirubin encephalopathy. A recent study reported an accuracy analysis of TCB measurements at uncovered skin sites with an interval of 2 h postphototherapy [12]. However, the mean differences between the TCB and TSB levels varied widely from -2.9 to -6.7 mg/dL in their study. The authors suggested that the TCB measurement was not suitable for neonates after phototherapy, even when measurements were recorded at covered sites and that measurement of TSB levels was necessary to ensure the selection of the appropriate treatment plan. In the study by Lucanova et al. [12], the TCB data from the lower abdominal skin sites covered with a diaper were chosen for comparison with other uncovered sites at the forehead, sternum and abdomen. They found that even for measurements at the covered area, the reliability was inadequate. In our study, the forehead skin site covered by a gauze eye mask was adopted as the best measurement area. Based on the mean differences and correlations with the corresponding TSB level, the TCB measurement at the forehead at 30 min postphototherapy was the most accurate among the measurements tested. The skin depth, subcutaneous tissue and composition are different between the forehead and lower abdomen, which may explain the discrepancies in results between the study by Lucanova et al. [12] and our study.

Different sites of transcutaneous bilirubin measurement have been reported, including the forehead, sternum, interscapular space, hipbone and abdomen [18]. The forehead and sternum are the most common uses in real clinical practice. Although the previous study has pointed out that the forehead could be more appropriate for TCB measurement [11], most other studies did not discuss the effect of the measurement intervals after phototherapy and whether the measurement site was covered or not [10]. In the study conducted in South Korea [11], the authors assessed TCB measurement within one hour after or before blood sampling for TSB. Their assessment timing for TCB measurement differed from our study, and these minor differences in study methods might lead to different results. A standardized protocol for TCB measurement can help to reach a consistent comparison between studies in the future. TcB measurement on covered skin on the forehead while applying eye protection during phototherapy revealed a better correlation with serum bilirubin levels [12,25]. Recently, a meta-analysis investigating the impact of phototherapy on the accuracy of TCB measurement showed only a moderate correlation between the TCB and TSB levels during phototherapy, and TCB measurement accuracy was improved postphototherapy [10]. Zecca et al. [20] also reported that the TCB measurement was more reliable at the covered area than at the uncovered skin sites during phototherapy. Another study by Murli et al. [26] reported that the agreement between the TCB and TSB levels was poor, that the TCB levels measured at covered areas may be overestimates relative to the TSB level and that TCB levels measured at uncovered areas may be underestimates relative to the TSB level at 12 h postphototherapy. Therefore, our study aimed to identify an easier and more accurate method of TCB measurement in neonates receiving phototherapy. We found that TCB measurement on the forehead at 30 min postphototherapy could be a good option because this method is a more convenient approach for clinical professionals to promptly evaluate neonatal jaundice without delayed management.

In prematurity, several reports, including meta-analysis research, reported reliable estimation results of transcutaneous bilirubin measurement of serum bilirubin levels, which are similar to our results [2,10]. However, there are still some suggestions for serum total bilirubin measurement to confirm transcutaneous results after the initiation of phototherapy [27]. One study pointed out that the interscapular site might be more appropriate for TCB measurement in preterm infants of 23 to 29 weeks of gestational age. In addition, it is necessary to be more cautious about the accuracy of TCB measurement in prematurity [28]. The threshold for phototherapy against neonatal hyperbilirubinemia is relatively low. If the TCB measurement underestimates the TSB data, it will misjudge the need for phototherapy [27]. The immaturity of skin in preterm infants and the absence of subcutaneous fat may be related to the rapid clearing of extravascular bilirubin under phototherapy [29,30]. Different albumin-to-bilirubin binding capacities in prematurity may also play roles in the lower accuracy of transcutaneous bilirubinometers [31].

Further clinical implications should consider different populations based on different races or other disease conditions. Overestimation of the total serum bilirubin level during transcutaneous bilirubin measurement has been reported, with an average difference of 0.67 mg/dL among African American newborns compared with other races [32]. One study in Nigeria also reported that transcutaneous bilirubin measurement was higher than total serum bilirubin by 2 to 3 mg/dL on average [33]. In neonates with hemolytic disease, transcutaneous bilirubin may be underestimated due to a rapid increase in serum total bilirubin [34]. In Japan, previous studies have reported a significant correlation in term infants and very low birth weight neonates [8,35].

Implications of transcutaneous bilirubin measurement for replacing serum bilirubin examination can reduce excessive piercing and blood sampling in neonates, which decrease the risk of infection and anemia in prematurity and avoid causing pain to the babies [36]. Transcutaneous screening of neonatal hyperbilirubinemia is also a convenient and cost-effective tool. One study in Pakistan with the implementation of transcutaneous screening protocols reported saving $1800 in six months [37]. For clinical implications with outliers, we should correct bilirubin levels while obtaining total serum bilirubin levels. To avoid underestimation, one systemic review suggested checking total serum bilirubin if the transcutaneous bilirubin level is higher than the phototherapy threshold minus 2.9 mg/dL [23].

### Strength and Limitations

The main strength of our study is that we compared four TCB measurements at different sites and time points after phototherapy with a concurrently obtained standard TSB measurement. Other studies compared TCB and TSB measurements but not in the same series. Compared with other studies, our study indicated a more convenient skin site (forehead) and evidence of a shorter time interval for TCB measurement postphototherapy. Our findings could help clinical practitioners better judge and avoid underestimating the serum bilirubin level.

This study has a few limitations. First, the majority of our study participants were late preterm and term infants. The applicability of these results may be limited to extremely preterm infants. Second, the range of the TSB levels was approximately 4.1–19.7 mg/dL; therefore, for TSB levels outside this range, the accuracy of the correlation between TCB and TSB measurements is uncertain. Third, we used handmade eye masks composed of cotton gauze to cover the measurement skin site, and the transparency of light and the coverage area was uncertain. No validation study was conducted to determine the true effect of blue light on the gauze-covered area. However, Rylance et al. [3] reported a good correlation between the TCB and TSB measurements in a study conducted in Malawi, and they applied a bulky gauze patch for eye protection and coverage of skin sites, similar to our method. Further investigation evaluating the efficacy and safety of gauze eye patches is needed.

## 5. Conclusions

Phototherapy may interfere with the accuracy of TCB measurement in neonates. Our findings suggest that the TCB levels measured on the forehead at 30 min postphototherapy had the strongest correlation with the corresponding TSB levels among the TCB measurements tested. The appropriate application of transcutaneous bilirubinometry could aid clinical practice and avoid unnecessary management. The accuracy of TCB measurement in extremely preterm infants needs to be more carefully interpreted. Future studies are needed to develop treatment guidelines for the use of TCB measurements and to assess the reliability of TCB measurements in extremely preterm infants.

## Figures and Tables

**Table 1:** Clinical characteristics of our study population.

Demographic Variables	Total	Term	Preterm
	*n* = 40	*n* = 27	*n* = 13
Gender (*n*, %)			
Male	24 (60)	14 (52)	10 (77)
Female	16 (40)	13 (48)	3 (23)
Gestational age, weeks (means ± SD)	37.2 ± 2.4	38.6 ± 0.9	34.3 ± 1.8
Birth weight, grams (means ± SD)	2920.7 ± 680	3235.4 ± 480	2267 ± 563
Small for gestational age (*n*,%)	3 (7.5)	0	3 (23)
Total bilirubin measures (*n*)	96	63	33
Transcutaneous bilirubin measures (*n*)	384	252	132
Perinatal factors			
Cesarean delivery (*n*, %)	8 (20)	3 (11)	5 (38)
Maternal preeclampsia (*n*, %)	5 (12.5)	2 (7)	3 (23)
Maternal gestational DM (*n*, %)	4 (10)	3 (11)	1 (7)

**Table 2:** Correlation and differences between TCB measurements among different skin sites and intervals after stopping phototherapy compared with TSB data.

Skin Sites and Interval	Mean/SD	Range	Mean of (TSB-TCB)	*r*
Forehead, 0 min	11.6/2.7	4.1~17.5	1.3	0.53
Forehead, 30 min	11.9/2.6	6.8~18.2	0.9	0.65
Mid-sternum, 0 min	7.1/3.4	0.7~16.6	5.8	0.52
Mid-sternum, 30 min	7.6/3.2	1.3~15.9	5.3	0.60

**Table 3:** Correlation and differences between TCB measurements among different skin sites and intervals after phototherapy compared with TSB data in term and preterm babies.

Skin Sites and Interval	Mean/SD	Range	Mean of (TSB-TCB)	*r*
Term				
Forehead, 0 min	11.5/2.7	4.1~16.4	1.7	0.50
Forehead, 30 min	11.5/2.5	7.8~18.2	1.2	0.64
Mid-sternum, 0 min	7.2/3.6	0.7~15.8	5.9	0.47
Mid-sternum, 30 min	7.6/3.3	1.3~15.9	5.6	0.60
Preterm				
Forehead, 0 min	11.8/2.7	5.8~17.5	0.5	0.67
Forehead, 30 min	12.1/2.7	6.8~17.0	0.3	0.69
Mid-sternum, 0 min	6.9/3.3	2.1~16.6	5.4	0.64
Mid-sternum, 30 min	7.7/2.9	2.8~14.0	4.7	0.62

**Table 4:** Distribution of differences between TCB and TSB measurements.

Skin Sites and Interval	Forehead 0 min	Forehead 30 min	Mid-Sternum, 0 min	Mid-Sternum, 30 min
2 > TCB − TSB	9(9.4%)	7(7.3%)	1(1%)	1(1%)
2 > TCB − TSB >−2	52(54.2%)	60(62.5%)	14(14.6%)	8(8.3%)
TCB − TSB <−2	35(36.4%)	29(30.2%)	81(84.4%)	87(90.7%)

## Data Availability

The data presented in this study are available on request from the corresponding author. The data are not publicly available due to patient confidentiality.

## Supplementary Materials

The following are available online at https://www.mdpi.com/article/10.3390/diagnostics11091729/s1, Table S1. Criteria of phototherapy; Figure S1. Schematic diagram of transcutaneous bilirubin measurement sites; Figure S2. Bland–Altman plot for evaluation agreement between TCB and TSB measurements in term babies after phototherapy. (**a**) Forehead (0 min), (**b**) Forehead (30 min), (**c**) Mid-sternum (0 min) and (**d**) Mid-sternum (30 min); Figure S3. Bland–Altman plot for evaluation agreement between TCB and TSB measurements in preterm babies after phototherapy. (**a**) Forehead (0 min), (**b**) Forehead (30 min), (**c**) Mid-sternum (0 min) and (**d**) Mid-sternum (30 min).

## References

[1] Bhutani V.K., Stark A.R., Lazzeroni L.C., Poland R., Gourley G.R., Kazmierczak S., Meloy L., Burgos A.E., Hall J.Y., Stevenson D.K. (2013). Predischarge screening for severe neonatal hyperbilirubinemia identifies infants who need phototherapy. J. Pediatr..

[2] Bhutani V.K., Zipursky A., Blencowe H., Khanna R., Sgro M., Ebbesen F., Bell J., Mori R., Slusher T.M., Fahmy N. (2013). Neonatal hyperbilirubinemia and Rhesus disease of the newborn: Incidence and impairment estimates for 2010 at regional and global levels. Pediatr. Res..

[3] Rylance S., Yan J., Molyneux E. (2014). Can transcutaneous bilirubinometry safely guide phototherapy treatment of neonatal jaundice in Malawi?. Paediatr. Int. Child Health.

[4] Samiee-Zafarghandy S., Feberova J., Williams K., Yasseen A.S., Perkins S.L., Lemyre B. (2014). Influence of skin colour on diagnostic accuracy of the jaundice meter JM 103 in newborns. Arch. Dis. Child Fetal Neonatal Ed..

[5] Wainer S., Rabi Y., Parmar S.M., Allegro D., Lyon M. (2009). Impact of skin tone on the performance of a transcutaneous jaundice meter. Acta Paediatr..

[6] Tan K.L., Dong F. (2003). Transcutaneous bilirubinometry during and after phototherapy. Acta. Paediatr..

[7] Katayama Y., Enomoto M., Kikuchi S., Takei A., Ikegami H., Minami H., Lee Y.K. (2017). Transcutaneous bilirubin measurement during phototherapy in term neonates. Pediatr. Int..

[8] Kurokawa D., Nakamura H., Yokota T., Iwatani S., Morisawa T., Katayama Y., Sakai H., Ioroi T., Iijima K., Morioka I. (2016). Screening for hyperbilirubinemia in Japanese very low birthweight infants using transcutaneous bilirubinometry. J. Pediatr..

[9] Radfar M., Hashemieh M., Shirvani F., Madani R. (2016). Transcutaneous bilirubinometry in preterm and term newborn infants before and during phototherapy. Arch. Iran Med..

[10] Nagar G., Vandermeer B., Campbell S., Kumar M. (2016). Effect of Phototherapy on the Reliability of Transcutaneous Bilirubin Devices in Term and Near-Term Infants: A Systematic Review and Meta-Analysis. Neonatology.

[11] Jeon J., Lim G., Oh K.W., Lee N.M., Park H.W., Chung M.L. (2020). The forehead is a better site than the sternum to check transcutaneous bilirubin during phototherapy in sick infants. BMC Pediatr.

[12] Casnocha Lucanova L., Matasova K., Zibolen M., Krcho P. (2016). Accuracy of transcutaneous bilirubin measurement in newborns after phototherapy. J. Perinatol..

[13] Hannemann R.E., DeWitt D.P., Wiechel J.F. (1978). Neonatal serum bilirubin from skin reflectance. Pediatr. Res..

[14] Hannemann R.E., Dewitt D.P., Hanley E.J., Schreiner R.L., Bonderman P. (1979). Determination of serum bilirubin by skin reflectance: Effect of pigmentation. Pediatr. Res..

[15] Jnah A., Newberry D.M., Eisenbeisz E. (2018). Comparison of transcutaneous and serum bilirubin measurements in neonates 30 to 34 weeks' gestation before, during, and after phototherapy. Adv. Neonatal Care.

[16] Pendse A., Jasani B., Nanavati R., Kabra N. (2017). Comparison of transcutaneous bilirubin measurement with total serum bilirubin levels in preterm neonates receiving phototherapy. Indian Pediatr..

[17] Rohsiswatmo R., Oswari H., Amandito R., Sjakti H.A., Windiastuti E., Roeslani R.D., Barchia I. (2018). Agreement test of transcutaneous bilirubin and bilistick with serum bilirubin in preterm infants receiving phototherapy. BMC Pediatr..

[18] Agrawal G., Garg K., Sitaraman S., Sarna A. (2019). Comparison of diagnostic accuracy of different sites for transcutaneous bilirubin measurement in early preterm infants. Indian J. Pediatr..

[19] Johnson S.M., Vasu V., Marseille C., Hill C., Janvier L., Toussaint P., Battersby C. (2020). Validation of transcutaneous bilirubinometry during phototherapy for detection and monitoring of neonatal jaundice in a low-income setting. Paediatr. Int. Child Health.

[20] Zecca E., Barone G., De Luca D., Marra R., Tiberi E., Romagnoli C. (2009). Skin bilirubin measurement during phototherapy in preterm and term newborn infants. Early Hum. Dev..

[21] Itoh S., Okada H., Kuboi T., Kusaka T. (2017). Phototherapy for neonatal hyperbilirubinemia. Pediatr. Int..

[22] Juster-Reicher A., Flidel-Rimon O., Rozin I., Shinwell E.S. (2015). Correlation of transcutaneous bilirubinometry (TcB) and total serum bilirubin (TsB) levels after phototherapy. J. Matern. Fetal Neonatal Med..

[23] Nagar G., Vandermeer B., Campbell S., Kumar M. (2013). Reliability of transcutaneous bilirubin devices in preterm infants: A systematic review. Pediatrics.

[24] Bhutani V.K., Johnson L.H., Gourley G. (2003). Measuring bilirubin through the skin?. Pediatrics.

[25] Fonseca R., Kyralessa R., Malloy M., Richardson J., Jain S.K. (2012). Covered skin transcutaneous bilirubin estimation is comparable with serum bilirubin during and after phototherapy. J. Perinatol..

[26] Murli L., Thukral A., Sankar M.J., Vishnubhatla S., Deorari A.K., Paul V.K., Sakariah A., Dolma, Agarwal R. (2017). Reliability of transcutaneous bilirubinometry from shielded skin in neonates receiving phototherapy: A prospective cohort study. J. Perinatol..

[27] Jegathesan T., Campbell D.M., Ray J.G., Shah V., Berger H., Hayeems R.Z., Sgro M. (2021). Transcutaneous versus Total Serum Bilirubin Measurements in Preterm Infants. Neonatology.

[28] Weber J., Vadasz-Chates N., Wade C., Micetic B., Gerkin R., Rao S. (2021). Transcutaneous Bilirubin Monitoring in Preterm Infants of 23 to 34 Weeks' Gestation. Am. J. Perinatol..

[29] Ozkan H., Duman N., Tuzun F. (2019). Dermal bilirubin kinetics during phototherapy in term neonates. J. Perinat. Med..

[30] Kanti V., Bonzel A., Stroux A., Proquitté H., Bührer C., Blume-Peytavi U., Bartels N.G. (2014). Postnatal maturation of skin barrier function in premature infants. Skin Pharmacol. Physiol..

[31] Karen T., Bucher H.U., Fauchère J.C. (2009). Comparison of a new transcutaneous bilirubinometer (Bilimed) with serum bilirubin measurements in preterm and full-term infants. BMC Pediatr..

[32] Taylor J.A., Burgos A.E., Flaherman V., Chung E.K., Simpson E.A., Goyal N.K., Von Kohorn I., Dhepyasuwan N. (2015). Discrepancies between transcutaneous and serum bilirubin measurements. Pediatrics.

[33] Olusanya B.O., Imosemi D.O., Emokpae A.A. (2016). Differences between transcutaneous and serum bilirubin measurements in black African neonates. Pediatrics.

[34] Mantagou L., Fouzas S., Skylogianni E., Giannakopoulos I., Karatza A., Varvarigou A. (2012). Trends of transcutaneous bilirubin in neonates who develop significant hyperbilirubinemia. Pediatrics.

[35] Yamana K., Morioka I., Kurokawa D., Fukushima S., Nishida K., Ohyama S., Nishimura N., Nozu K., Taniguchi-Ikeda M., Nagase H. (2017). Evaluation of BiliCare transcutaneous bilirubin device in Japanese newborns. Pediatr. Int..

[36] Counsilman C.E., Heeger L.E., Tan R., Bekker V., Zwaginga J.J., Te Pas A.B., Lopriore E. (2021). Iatrogenic blood loss in extreme preterm infants due to frequent laboratory tests and procedures. J. Matern. Fetal Neonatal Med..

[37] Shah M.H., Ariff S., Ali S.R., Chaudhry R.A., Lakhdir M.P.A., Qaiser F., Demas S., Hussain A.S. (2019). Quality improvement initiative using transcutaneous bilirubin nomogram to decrease serum bilirubin sampling in low-risk babies. BMJ Paediatr Open.

