# Supplementary material for: The Impact of Phototherapy on the Accuracy of Transcutaneous Bilirubin Measurements in Neonates: Optimal Measurement Site and Timing
Source: Diagnostics (Basel). 2021 Sep 20;11(9):1729. doi: 10.3390/diagnostics11091729 (PMC8466921; doi:10.3390/diagnostics11091729)
Supplement: Supplementary file 1 [file diagnostics-11-01729-s001.zip › diagnostics-1337669-supplementary.pdf]

Supplementary information

Table S1. Criteria of phototherapy.

1. Criteria of Phototherapy

Days / Body weight/weeks	0.5	1	2	3	4	≥ 5
<1000 gm	4	6	6	8	8	8
1000-2000 gm	6	8	10	12	12	12
2001-2499gm or GA 35-36 $^{6/7}$ weeks	6	8	11	13	14	15
≥ 2500gm and GA ≥ 37 weeks	7.8	9.9	13.1	15.5	17.5	18

2. Criteria of Intensive phototherapy

Days / Body weight/weeks	0.5	1	2	3	4	≥ 5
<1000 gm	6	8	8	10	10	10
1000-2000 gm	7	9	11	13	15	15
2001-2499gm or GA 35-36 $^{6/7}$ weeks	8	10	13	15	17	18
≥ 2500gm and GA ≥ 37 weeks	9	12	15	18	20	21

3. Criteria of blood exchange transfusion

Days / Body weight/weeks	0.5	1	2	3	4	≥ 5
<1000 gm	8	10	10	12	13	15
1000-2000 gm	10	12	15	17	17	17
2001-2499gm or GA 35-36 $^{6/7}$ weeks	13	14	17	21	21	21
≥ 2500gm and GA ≥ 37 weeks	15	16.5	19	24	25	25

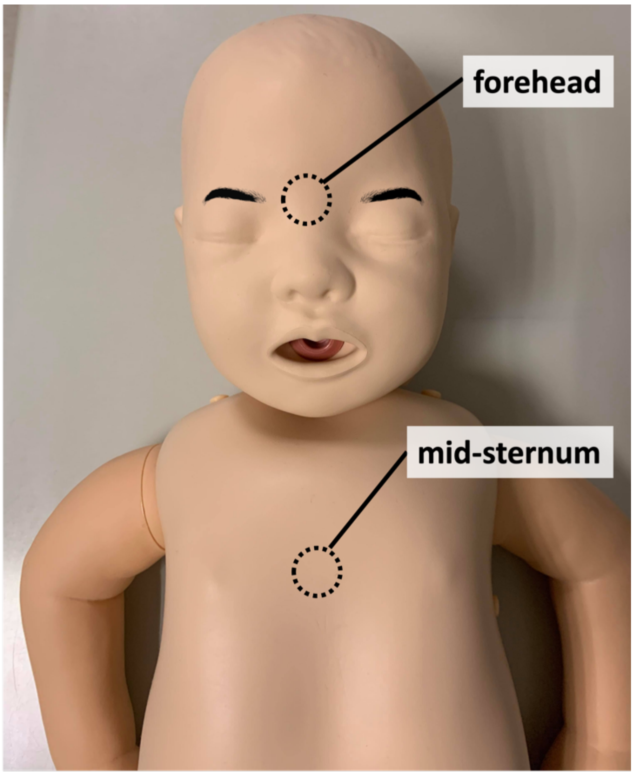

Figure S1. Schematic diagram of transcutaneous bilirubin measurement site.

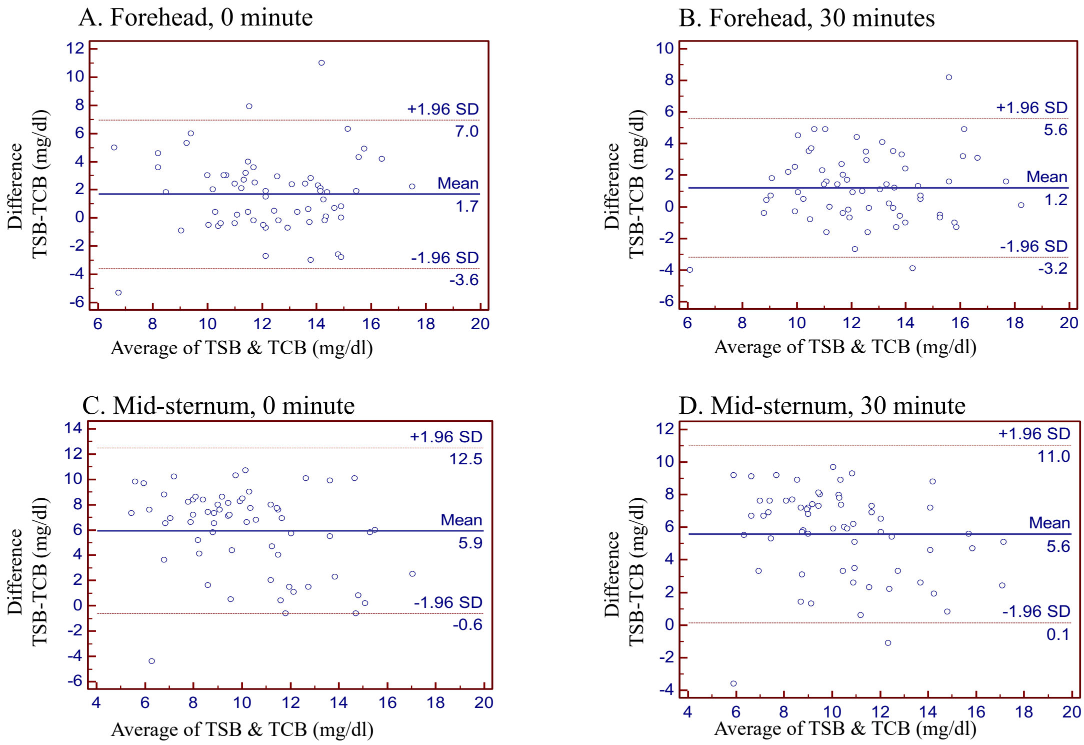

Figure S2. Bland-Altman plot for evaluation agreement between TCB and TSB measurements in term babies after phototherapy. (**a**) Forehead (0 min), (**b**) Forehead (30 mins), (**c**) Mid-sternum (0 min) and (**d**) Mid-sternum (30 mins).

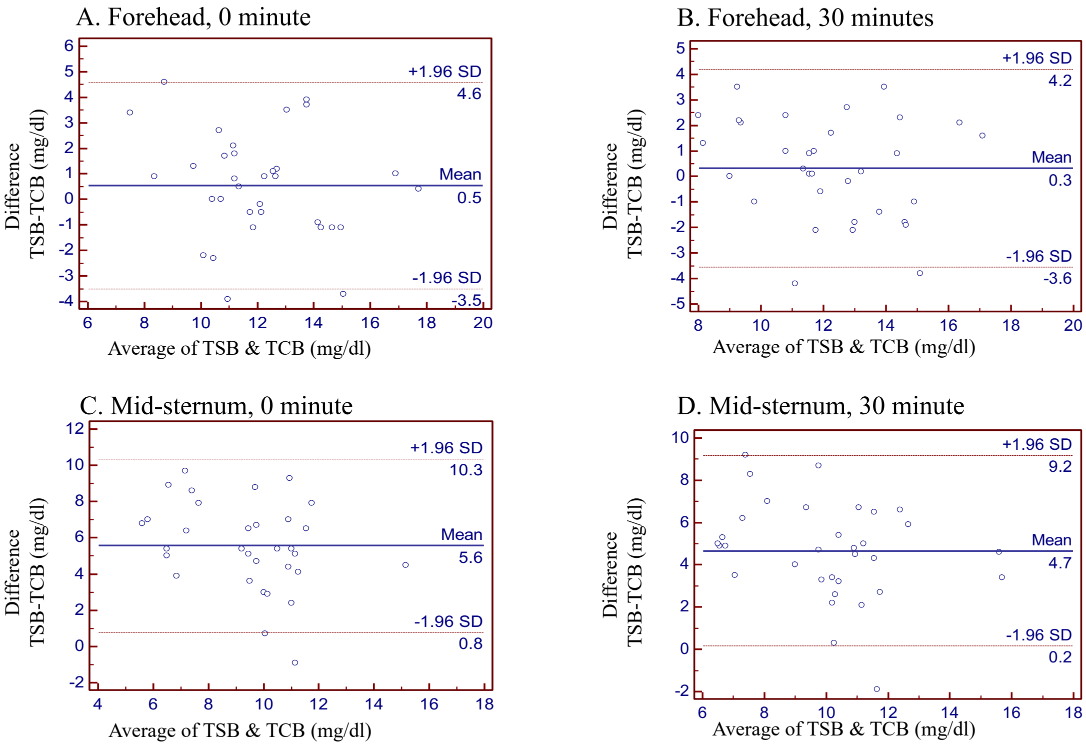

Figure S3. Bland-Altman plot for evaluation agreement between TCB and TSB measurements in preterm babies after phototherapy. (**a**) Forehead (0 min), (**b**) Forehead (30 mins), (**c**) Mid-sternum (0 min) and (**d**) Mid-sternum (30 mins).
